# Body image and weight control in South Africans 15 years or older: SANHANES-1

**DOI:** 10.1186/s12889-015-2324-y

**Published:** 2015-09-30

**Authors:** Zandile J. Mchiza, Whadi-ah Parker, Mokhantso Makoae, Ronel Sewpaul, Takura Kupamupindi, Demetre Labadarios

**Affiliations:** Population Health, Health Systems and Innovation (PHHSI), Human Sciences Research Council, Private Bag X9182, Cape Town, 8000 12th Floor, Plein Park Building, 69-83 Plein Street, Cape Town, 8001 South Africa; Human and Social Development, Human Sciences Research Council, Cape Town, South Africa

**Keywords:** Body image, Weight status, Ethnicity, Dichotomy of body image, Body image distortion, Body image dissatisfaction

## Abstract

**Background:**

South African studies have suggested that differences in obesity prevalence between groups may be partly related to differences in body image and body size dissatisfaction. However, there has never been a national study that measured body image and its relationship to weight control in the country. Hence, the main aim of the study was to examine body image in relation to body mass index and weight control in South Africa.

**Methods:**

A cross-sectional survey and a secondary analyses of data were undertaken for 6 411 South Africans (15+ years) participating in the first South African National Health and Nutrition Examination Survey. Body image was investigated in relation to weight status and attempts to lose or gain weight. Data were analysed using STATA version 11.0. Descriptive statistics are presented as counts (numbers), percentages, means, standard error of means, and 95 % confidence intervals. Any differences in values were considered to be significantly different if the confidence intervals did not overlap.

**Results:**

Overall, 84.5 % participants had a largely distorted body image and 45.3 % were highly dissatisfied about their body size. Overweight and obese participants under estimated their body size and desired to be thinner. On the other hand, normal- and under-weight participants over estimated their body size and desired to be fatter. Only 12.1 and 10.1 % of participants attempted to lose or gain weight, respectively, mainly by adjusting dietary intake and physical activity.

**Discussion:**

Body mass index appears to influence body image and weight adjustment in South Africa.

**Conclusions:**

South Africans at the extreme ends of the body mass index range have a largely distorted body image and are highly dissatisfied by it. This suggests a need for health education and beneficial weight control strategies to halt the obesity epidemic in the country.

## Background

The prevalence of overweight and obesity has consistently been reported to have risen, albeit variably, across world regions and populations as a result of a prevailing “obesogenic” environment [1, 2]. The determinants of obesity in a developing country such as South Africa need to be better understood. Although globalisation and urbanisation are considered to be major drivers of the emerging epidemic, the aetiology of obesity is complex [3]. In addition to the biology of individuals [4], there are behavioural determinants [5], along with economic [6], environmental and socio-cultural factors [7]. These factors either directly or indirectly influence the development of obesity. There are gaps in our knowledge regarding socio-cultural determinants of obesity and body image (BI), in particular at a national level [8, 9].

Various international studies suggest that BI is multidimensional [10]. The dimensions of BI include issues relating to body size perception (meaning the way people see their body size) [11] and attitudes (meaning how people feel about their body size) [12]. These dimensions are thought to determine whether an individual will prefer to be thin or fat. Moreover, they promote behaviours that include adjusting energy intake and energy expenditure. Notably, the afore-mentioned effects appear to be remarkably different across culture, age, gender and social class [7, 13–18].

In the BI literature, researchers compare measured and estimated body mass index (BMI) to determine body image distortion [10]. They then compare perceived body size with an individual’s ideal body size to determine the degree of satisfaction with body size [12]. Because BI can be considered as a visual phenomenon, researchers tend to use different forms of figural scales to assess BI perception and attitudes [19–22]. The typical scales include asking respondents to identify the figure that most closely resembles their current body size as they “Feel” or perceive it, as well as the figure that most closely resembles what they would regard as their “Ideal” body size. The most commonly used versions of these scales include nine line drawings that are gender-specific [21] and BMI specific [23], ranging from the very thin to the obese, arranged in ascending order of body size.

The afore-mentioned methodologies have been adopted and validated for use with different ethnicities and age groups in South Africa [15]. However, to our knowledge no national study has investigated the different dimensions of BI in relation to measured weight status (BMI) and weight management practices, which is the purpose of this study.

## Methods

### Study design and study population

This current cross sectional study employed secondary analyses of data for 6 411 South Africans aged 15 years and older who took part in the first South African National Health and Nutrition Examination Survey (SANHANES-1) [24]. The SANHANES is a survey that measures the nutrition and health status of South Africans and was conducted by the Human Sciences Research Council (HSRC) in 2012. The survey was designed to yield a representative sample of residents of South Africa.

According to the 2011 Census [25], South Africa is a multi-ethnic country with a total population of 51 770 560, where the people are concentrated in two of the nine provinces, namely Gauteng (23.7 %, N = 12 272 263) and KwaZulu Natal (19.8 %, N = 10 267 300) [25]. The South African population has a slight excess of females (51.3 % females vs 48.7 % males), while the majority are black (79.2 %), followed by white (8.9 %), coloured (8.9 %) and then Indian and Asian (2.5 %) races [25]. In this round of analysis, data was standardized (weighted) to represent the South African ethnic diversity, geographic location (in terms of provinces, and urban/rural area of residence) and gender, based on the 2011 Census [25].

### Sampling and recruitment

The survey applied a multi-stage disproportionate, stratified cluster sampling approach. Enumeration areas (EAs) or groups of dwellings, within the area of research interest were used as the primary sampling units. Ten thousand households were selected from the 500 selected EAs. Of the 10 000 households, 8 166 households were found to be valid, occupied households; with the rest being abandoned households. Within each household, all members were eligible to participate in the survey. The 8 166 households yielded 8 776 eligible individuals, 15 years or older who consented to answer questions for the 2012 SANHANES-1 BI survey. Of these 8 776 individuals, only 6 411 had valid recorded weight and height readings as well as valid calculated BMI and complete answers to the questions regarding BI, and were therefore included in the current secondary analyses.

### Anthropometric variables

Body weight and height of the 6 411 participants were assessed using the techniques described by Lee and Nieman [26] and BMI was calculated for all participants as weight (in kilograms) divided by the square of height (in metres) and expressed as kg/m^2^. The recommended Centers for Disease Control (CDC) BMI-for-age (indicated as a percentile) [27] and BMI (indicated as kg/m^2^) [28] cut-offs for children (15-18 year olds) and adults (19+ year olds), respectively were used. Underweight was defined as BMI < 5^th^ percentile and BMI < 18.5 kg/m^2^ for children and adults, respectively. Normal weight was defined as BMI = 5^th^ - 84.9^th^ percentile as well as BMI = 18.5 kg/m^2^ - 24.9 kg/m^2^ for children and adults, respectively. Overweight was defined as BMI = 85^th^ - 94.9^th^ percentile; and BMI = 25 kg/m^2^ - 29.9 kg/m^2^ for children and adults, respectively. Finally, obesity was defined as BMI ≥ 95^th^ percentile and BMI ≥ 30 kg/m^2^ for children and adults, respectively [27, 28].

### Questionnaire

An age- and ethnicity-adjusted validated questionnaire, examining BI perception, BI ideals, as well as weight-related behaviours was used [15]. Additional information was collected on socio-demographic factors such as age, ethnicity, gender, geographic location, socioeconomic status (defined on the basis of the household income), marital status and education level. The figural stimuli used to identify body size and shape status of males and females were based on nine silhouettes [21]. These silhouettes were previously used successfully in adults and adolescent children in South Africa [15, 16, 29]. These silhouettes were also allocated BMI coordinates (*i.e.* mean BMI and BMI percentiles) ranging from 18-45 kg/m^2^ and the 4^th^ – 96^th^ BMI percentiles for adults and children, respectively [23].

### Body image dissatisfaction

To assess BI dissatisfaction using the Feel Ideal Difference (FID) index [15], participants were requested to identify the silhouette that most closely resembled their current body size “Feel”, as well as the silhouette they ideally wanted to look like or regarded as “Ideal”. The difference between the scores for “Feel” and “Ideal” were then calculated to determine the FID index score (*i.e. FID index score = Score for “Feel” – Score for “Ideal”*). The FID index score could either be positive showing the desire to be thinner or negative showing the desire to be bigger. A FID index score of zero indicated congruency between the “Feel” and “Ideal” scores. A FID index score that was closer to zero represented less body size dissatisfaction; whereas, a FID index score that was lower than -1 or higher than +1 indicated greater body size dissatisfaction.

### Body image distortion

The selected “Feel” silhouette was also allocated a BMI/BMI percentile coordinate, regarded as estimated BMI/BMI percentile corresponding to the number of the selected silhouette [23]. The BMI/BMI percentile coordinate was then subtracted from the actual calculated BMI/BMI percentile to generate a score that identified the BI distortion (BID index score) for each participant (*i.e. BID index score = calculated BMI – estimated BMI)*. The BID index score could either be positive showing underestimation of body size or negative showing overestimation of body size. A BID index score of zero indicated congruency between the calculated BMI/BMI percentile and the estimated BMI/BMI percentile. Since the BMI coordinates [23] used were not absolute values because they represent BMI/BMI percentile ranges, BID index scores that were closer to zero represented more-or-less correct estimation of body size; whereas, BID index scores that were lower than -1 or higher than +1 indicated larger BI distortion.

### Weight-related behaviours

In addition, participants were asked whether they had ever attempted to lose or gain weight. If their response was “yes”, they were asked to identify the methods used for weight loss or gain and, the factors that contributed to their weight loss or gain.

### Ethical issues

SANHANES-1 received ethics approval from the Research Ethics Committee of the Human Science Research Council (HSRC) of South Africa (REF: REC6/16/11/11). All survey participants signed informed consent forms which had been explained to them. In addition, parent/guardians of children aged 1 to 17 years signed consent forms which were explained to them on behalf of their children to allow them to participate in the survey. Permission to use the SANHANES-1 data was obtained from Prof Demetre Labadarios (the Executive Director of the HSRC’s Population Health, Health Systems and Innovation [PHHSI] programme, and a Principal Investigator of the survey).

### Statistical analysis

Analysis was completed using STATA version 11.0 (StataCorp, 2009) and Microsoft Excel. The “svy” method in STATA was used to create estimates that adjust for the complex, multi-level sampling design. In the current analyses, the survey sample was stratified by province and enumerator areas. In this regard “svy” was used to account for unequal sampling probabilities in order to benchmark (standardise) the sample to represent the South African Census 2011 [25] population estimates. Weighted data were analysed using univariate and bivariate analysis techniques. Mean BMI and BMI percentiles were used to demonstrate the prevalence of underweight, normal weight, overweight and obesity. The mean BID and FID index scores were also reported based on the body weight. Estimates (in counts [number] and prevalence rates [percentages]) were reported. Furthermore, the prevalence of attempts to lose or gain weight and the methods of weight loss or gain were reported as percentages based on the FID index score categories. Estimates (means and prevalence rates) were reported with corresponding 95 % confidence intervals (CI) and standard error of means. Any differences in CI values were considered to be significant if they did not overlap.

## Results

### The socio-demographic profile and anthropometric status

Socio-demographic and anthropometric details of the larger SANHANES-1 sample are outlined in a report by Shisana *et al.* [24]. Of the 6 411 participants included in this analysis (Table 1), the majority 57.6 % were female, 19-24 years (16.5 %), black South Africans (82.4 %), resided in urban formal settings (51.1 %) located in Gauteng (24.7 %), never married (45.8 %), completed grades 8-11 (39.1 %) and earned no income (34.0 %) or had an income of between ZAR^1^ 9 601 and ZAR^i^38 400 (38.6 %) per annum.

**Table 1:** Socio-demographic characteristics and body mass index of South Africans aged 15 years or older, SANHANES-1, 2012 [24]

				Body mass index (BMI) ^b^							
	Sample size	BMI		Underweight		Normal weight		Overweight		Obese	
	n(%)^a^	Mean (SEM)^d^	95 % CI	%	95 % CI	%	95 % CI	%	95 % CI	%	95 % CI
Sex											
Males	2246(42.4)	23,88(-0,22)	[23.44-24.32]	11,6	[9.7-13.9]	55,9	[52.9-58.8]	20,5	[18.1-23.2]	12,0	[9.9-14.4]
Females	4165(57.6)	28,96(-0,21)	[28.55-29.37]	3,7	[3.0-4.6]	32,0	[29.9-34.1]	24,9	[23.0-26.9]	39,4	[37.0-41.9]
Age (years)											
15 – 18^b^	764(12.4)	21,86(-0,22)	[21.43-22.30]	14,0	[10.7-18.0]	65,1	[60.4-69.6]	15,7	[12.4-19.6]	5,2	[3.5-7.7]
19 – 24	985(16.5)	24,50(-0,29)	[23.93-25.07]	9,2	[6.0-13.9]	57,0	[52.3-61.6]	17,9	[14.9-21.2]	15,9	[13.2-19.1]
25 - 34	1082(15.7)	26,83(-0,29)	[26.27-27.39]	4,2	[3.0-5.7]	44,8	[40.8-48.9]	24,9	[21.3-28.8]	26,1	[22.6-30.0]
35 - 44	980(14.5)	27,82(-0,35)	[27.14-28.51]	6,3	[4.7-8.3]	38,1	[33.7-42.7]	22,1	[18.4-26.2]	33,6	[29.3-38.1]
45 - 54	1022(15.8)	29,16(-0,42)	[28.33-29.98]	5,1	[3.5-7.4]	29,6	[25.4-34.0]	26,0	[21.2-31.4]	39,3	[34.3-44.6]
55 - 64	856(13.5)	28,93(-0,50)	[27.96-29.91]	6,6	[4.6-9.2]	27,4	[22.7-32.7]	26,4	[22.6-30.5]	39,6	[34.5-45.0]
> = 65	722(11.5)	28,44(-0,42)	[27.62-29.26]	4,6	[3.3-6.4]	31,6	[27.3-36.3]	29,2	[24.9-33.8]	34,7	[30.0-39.6]
Race											
Black	4528(82.4)	26,83(-0,19)	[26.46-27.20]	6,9	[5.8-8.3]	42,5	[40.5-44.4]	22,7	[20.9-24.6]	27,9	[26.0-29.8]
White	135(3.3)	28,72(-1,00)	[26.75-30.69]	0,6	[0.1-3.0]	31,8	[20.0-46.5]	27,4	[17.9-39.5]	40,2	[27.4-54.5]
Mixed ancestry	1428(11.8)	26,44(-0,32)	[25.81-27.07]	7,0	[5.4-9.0]	43,3	[39.0-47.8]	24,0	[21.0-27.3]	25,7	[21.9-29.8]
Indian	299(2.6)	25,21(-1,42)	[22.42-28.00]	20,0	[8.4-40.7]	35,6	[28.7-43.2]	22,6	[13.2-35.9]	21,8	[12.0-36.1]
Locality											
Urban formal	3084(51.1)	27,46(-0,28)	[26.91-28.01]	5,8	[4.3-7.8]	39,2	[36.6-42.0]	24,1	[21.6-26.9]	30,9	[27.9-34.0]
Urban informal	788(9.9)	26,39(-0,41)	[25.58-27.21]	9,5	[5.7-15.4]	42,5	[37.2-48.0]	23,9	[20.0-28.2]	24,1	[20.6-28.0]
Rural informal (Tribal)	1621(30.5)	26,26(-0,23)	[25.80-26.72]	7,5	[6.0-9.4]	45,0	[42.4-47.7]	21,2	[19.2-23.4]	26,2	[24.0-28.5]
Rural formal (Farms)	918(8.5)	25,32(-0,32)	[24.69-25.94]	10,3	[8.2-12.8]	48,2	[43.5-52.9]	21,9	[16.8-28.2]	19,6	[16.4-23.2]
Province											
Western Cape	1052(12.3)	27,03(-0,37)	[26.31-27.76]	5,2	[3.8-7.1]	40,0	[35.5-44.8]	26,3	[22.6-30.4]	28,4	[23.8-33.5]
Eastern Cape	802(11.6)	26,39(-0,34)	[25.72-27.05]	7,4	[5.5-9.9]	44,0	[39.8-48.4]	20,0	[17.0-23.3]	28,6	[25.1-32.4]
Northern Cape	399(2.4)	26,27(-0,78)	[24.73-27.80]	9,4	[5.7-15.0]	45,0	[36.9-53.5]	20,2	[16.7-24.2]	25,5	[19.8-32.2]
Free State	468(5.7)	26,89(-0,49)	[25.93-27.85]	7,8	[5.7-10.7]	42,3	[35.0-49.9]	22,0	[18.3-26.2]	27,9	[23.5-32.7]
KwaZulu Natal	893(17.6)	27,13(-0,46)	[26.23-28.03]	7,9	[4.5-13.5]	39,3	[34.9-43.8]	22,7	[19.5-26.1]	30,2	[26.0-34.7]
North West	789(7.0)	24,81(-0,28)	[24.27-25.36]	13,0	[9.6-17.4]	49,1	[44.7-53.5]	16,3	[13.1-20.2]	21,5	[18.2-25.3]
Gauteng	739(24.7)	27,71(-0,47)	[26.79-28.64]	4,8	[2.8-8.0]	39,4	[35.2-43.8]	26,2	[21.8-31.1]	29,6	[24.8-34.9]
Mpumalanga	720(7.5)	26,63(-0,46)	[25.72-27.54]	6,0	[3.8-9.3]	45,3	[41.1-49.5]	22,6	[19.9-25.7]	26,1	[21.7-31.1]
Limpopo	549(11.1)	25,91(-0,32)	[25.29-26.53]	8,5	[6.3-11.5]	45,6	[40.9-50.4]	21,8	[17.3-27.0]	24,0	[20.7-27.8]
Marital status^c^											
Never married	2404(45.8)	25,26(-0,23)	[24.81-25.71]	9,1	[7.1-11.6]	50,3	[47.6-53.0]	20,1	[17.7-22.7]	20,5	[18.3-22.8]
Living together	462(8.0)	27,01(-0,45)	[26.12-27.90]	5,9	[4.0-8.5]	41,7	[35.5-48.2]	29,1	[23.5-35.4]	23,3	[18.4-29.0]
Married	1817(34.7)	28,75(-0,31)	[28.13-29.36]	3,9	[2.9-5.3]	31,4	[28.1-34.8]	27,3	[24.4-30.5]	37,4	[33.8-41.0]
Widowed/Separated/Divorced	639(11.5)	29,87(-0,53)	[28.84-30.91]	4,8	[3.0-7.4]	27,2	[22.8-32.0]	24,1	[20.1-28.6]	43,9	[38.2-49.8]
Highest level of Education											
No schooling	554(8.9)	26,94(-0,49)	[25.97-27.91]	12,4	[7.9-19.0]	34,0	[29.3-39.1]	23,3	[19.3-27.9]	30,2	[25.6-35.3]
Grades 0-5	645(11.1)	27,06(-0,50)	[26.08-28.03]	9,6	[6.8-13.5]	38,3	[34.0-42.9]	21,0	[16.7-26.1]	31,0	[25.9-36.6]
Grades 6-7	643(11.1)	27,10(-0,43)	[26.25-27.95]	7,7	[5.4-11.0]	38,9	[33.3-44.8]	25,8	[21.0-31.3]	27,6	[23.1-32.6]
Grades 8-11	2185(39.1)	26,38(-0,25)	[25.89-26.86]	7,3	[5.5-9.6]	45,4	[42.4-48.3]	21,3	[18.8-23.9]	26,1	[23.6-28.7]
Grade 12	1083(22.1)	26,70(-0,32)	[26.07-27.33]	4,6	[2.7-7.5]	46,8	[42.3-51.4]	23,2	[18.9-28.2]	25,4	[21.5-29.7]
Higher education	316(7.8)	28,35(-0,62)	[27.14-29.56]	1,2	[0.5-2.7]	31,4	[23.9-40.0]	31,8	[24.3-40.3]	35,6	[27.5-44.7]
Annual income category (ZAR^i^)^c^											
No income	1413(34.0)	26,51(-0,31)	[25.89-27.13]	8,2	[5.9-11.2]	46,6	[43.3-49.9]	18,9	[16.2-21.8]	26,3	[23.5-29.3]
1-9600	558(14.3)	27,51(-0,46)	[26.61-28.42]	7,0	[4.4-10.9]	36,6	[31.4-42.0]	22,5	[18.6-27.0]	33,9	[28.4-39.8]
9601-38400	1593(38.6)	27,77(-0,33)	[27.12-28.41]	5,9	[4.2-8.2]	37,7	[34.2-41.3]	24,7	[21.4-28.2]	31,8	[28.1-35.7]
38401-153600	320(9.8)	28,23(-0,52)	[27.21-29.24]	1,2	[0.5-2.8]	30,6	[22.6-40.1]	32,3	[23.4-42.7]	35,8	[28.0-44.5]
> = 153601	97(3.2)	29,13(-0,84)	[27.48-30.79]	0,2	[0.0-1.8]	25,0	[13.9-40.9]	33,5	[18.0-53.5]	41,3	[24.5-60.3]
Total	6411(100)	26,81(-0,17)	[26.47-27.14]	7,0	[6.0-8.3]	42,1	[40.4-43.9]	23,0	[21.4-24.7]	27,8	[26.1-29.6]

^a^Weighted percentage to represent the South African ethnic diversity, geographic location (in terms of provinces; urban/rural) and gender, based on the 2011 Census [25]

^b^BMI percentiles used to categorise weight status in children 15-18 years

^c^For persons 18 years and older

^d^SEM – Standard error of the mean

The overall mean BMI was 26.8 kg/m^2^, with 42.1 % of South Africans within the normal BMI range, while 23.0 % and 27.8 % were overweight and obese, respectively (Table 1). Only 7.0 % of the participants in this secondary analysis were classified as underweight. Females had a significantly higher mean BMI (29.0 kg/m^2^) than males (23.9 kg/m^2^). Nearly one third (32.5 %) of the males were overweight and obese compared to twice as many (64.3 %) females. By contrast, 11.6 % and 3.7 % of males and females were underweight, respectively. There was also a trend showing that the mean BMI increased with age, being highest in the 45-54 year (29.2 kg/m^2^) and 55-64 year age group (28.9 kg/m^2^), while the youngest age group (15-18 year olds) had a significantly lower mean BMI (21.9 kg/m^2^) than all other age groups. Less than a quarter (20.9 %) of the youngest age group were overweight (15.7 %) or obese (5.2 %) with 14.0 % underweight, while more than 30 % of participants in all other age groups were overweight or obese and less than 10 % were underweight. There were no significant differences in BMI by race.

The combined prevalence of overweight and obesity was highest in urban formal settings (55.0 %) compared to all other settings (range: 41.5-48.0 %). The mean BMI of participants in urban formal settings was significantly higher (27.5 kg/m^2^) compared to that of participants in rural informal (tribal areas) (26.3 kg/m^2^) and rural formal areas (farms) (25.3 kg/m^2^).

Participants in Gauteng, KwaZulu Natal and Western Cape (the most urbanized provinces) [25] had the highest mean BMI (27.7 kg/m^2^, 27.1 kg/m^2^ and 27.0 kg/m^2^, respectively). Participants in the North West (one of the more rural provinces) [25] had a significantly lower mean BMI (24.8 kg/m^2^) compared to all other provinces except the Northern Cape (26.3 kg/m^2^) and Limpopo (25.9 kg/m^2^) (also more rural provinces) [25]. The North West was the only province in which the combined prevalence of overweight and obesity was less than 40 %, while about half (45.8-55.8 %) of the participants in all the other provinces were overweight and obese.

With regard to marital status, participants who were never married had a significantly lower mean BMI (25.3 kg/m^2^) compared to those who were living together (27.0 kg/m^2^), married (28.8 kg/m^2^) or divorced/separated/widowed (29.9 kg/m^2^). More than two thirds of participants who were married (64.7 %) or divorced/separated/widowed (68.0 %) were overweight and obese, compared to 52.4 and 40.6 % of participants who lived with partners and those who were never married, respectively.

The only significant difference in mean BMI with regard to education was between the group that had higher education (28.4 kg/m^2^) and those that had completed grades 8-11 (26.4 kg/m^2^). However, more than two thirds (67.4 %) of participants in the group that had completed higher education were overweight and obese compared to about half (47.4-53.5 %) of all other participants.

In relation to income, the group that earned no income had a significantly lower mean BMI (26.5 kg/m^2^) compared to the groups that earned more than ZAR^i^38 401 annually. Close to half (45.2 %) of the participants in the no income group were overweight and obese, whereas 68.1 and 74.8 % of participants who earned ZAR^i^38 401 – ZAR^i^153 600 and more than ZAR^i^153 600, respectively were overweight or obese.

### The distribution of body image dissatisfaction and distortion

**Fig. 1:**
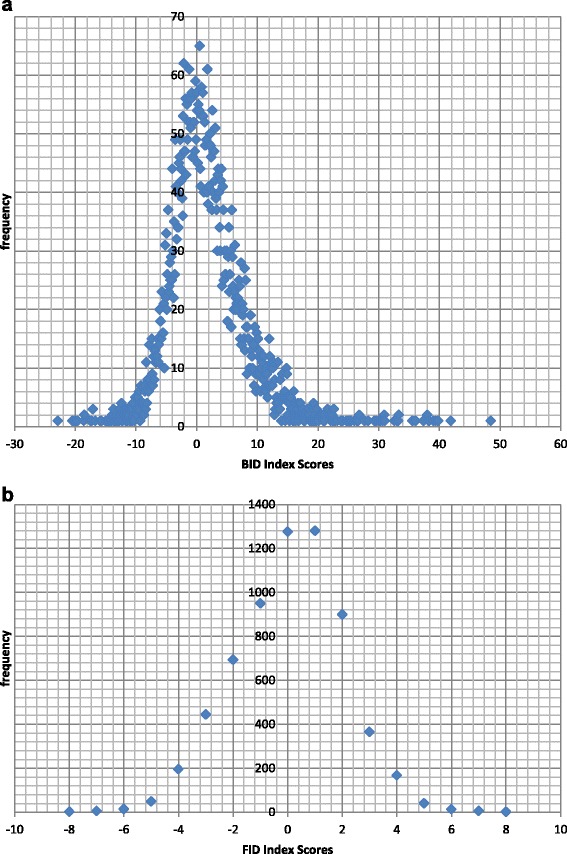
Frequency distribution of body image distortion (BID, **a**) and body image dissatisfaction (FID, **b**) index scores for adult South Africans aged 15 years and older, SANHANES 2012 [24]

Overall, the frequency of BID Index scores assumed a normal curve (Fig. 1a) with 49.8 % (N = 3195; BID Index scores = 1 to 48.5) falling on the positive side of the curve, indicating that nearly half the participants underestimated their BMI to a larger degree. Only 1 % (N = 57) of participants correctly estimated their body size since their BID Index scores fell on the zero axis, while 14.5 % (N = 933) of the participants’ data either fell between -1 and 0 or 0 and 1, an indication of close correct estimation of body size. The remaining 34.7 % of the results (N = 2226) were on the negative side of the curve (BID Index = -1 to -22.9), indicating that nearly 35 % of participants overestimated their BMI to a larger degree.

Moreover, the frequency of FID Index scores followed a normal curve (Fig. 1b) with 20 % (N = 1282) falling directly on the zero axis. This indicates that one fifth of participants were satisfied with their current weight. A further 19.9 % (N = 1280) and 14.8 % (N = 940) of the mean FID Index scores were clustered around zero, *i.e.* between 0 and +1 and 0 and -1, respectively. On the other hand, 23.4 % (N = 1501) obtained higher positive mean FID Index scores (ranging from 1 to 8), while 21.9 % (N = 1404) obtained negative mean FID Index scores (ranging from -1 to -8), suggesting that close to a quarter of participants ideally wanted to be either thinner or bigger, respectively.

### The direction and degree of body image dissatisfaction and distortion in relation to BMI categories

**Fig. 2:**
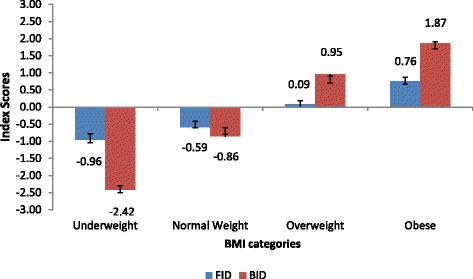
Mean index scores determining BI dissatisfaction (FID) and distortion (BID) in South Africans aged 15 years and older by different BMI categories, SANHANES 2012 [24]

According to BMI categories, underweight and normal weight participants had negative mean index scores for both FID and BID, indicating that they wanted to be fatter than how they perceived themselves and at the same time overestimated their body size (Fig. 2). Overweight and obese participants on the other hand had positive mean FID and BID index scores, indicating that they ideally wanted to be thinner than how they perceived themselves and at the same time underestimated their body size.

For both FID and BID, underweight participants had significantly higher negative mean index scores (-0.96 and -2.42, respectively) than normal weight participants (-0.59 and -0.86, respectively), indicating a larger degree of both dissatisfaction and distortion in underweight participants than their normal weight counterparts (Fig. 2). Similarly, obese participants had slightly higher positive mean FID and BID index scores (0.76 and 1.87 respectively) than overweight participants (0.09 and 0.95 respectively), indicating a larger degree of both dissatisfaction and distortion in obese participants than their overweight counterparts.

### The FID and BID index scores across BMI categories

**Fig. 3:**
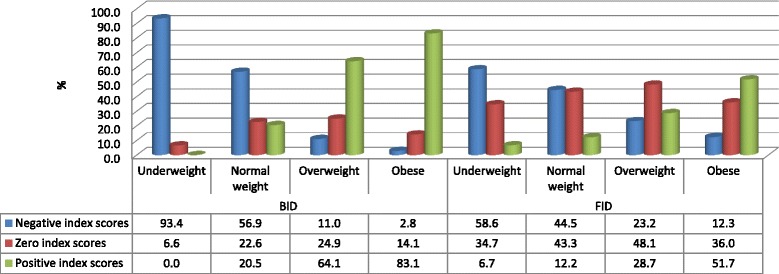
Prevalence of FID and BID index scores across BMI categories of South Africans aged 15 years and older who were respondents in the SANHANES-1 survey [24]

The same described trend was evident when comparing the direction and degree of dissatisfaction and distortion across BMI categories (Fig. 3). In this regard, for both FID and BID the prevalence of participants who achieved negative index scores tended to decrease as BMI increased. Conversely, the prevalence of participants who achieved positive index scores tended to increase as BMI increased. This implies that the majority of participants who perceived themselves to be thinner wanted to be bigger, while those who perceived themselves as fatter wanted to be thinner. It should also be noted that in this figure, a significant number of participants (more than a third) of varying BMI categories obtained FID index scores that were equal to zero, suggesting that they were satisfied with their body size. Similarly, more than a fifth of normal weight and overweight participants obtained BID index scores that were equal to zero, suggesting that they could estimate their body size correctly.

### Methods of weight loss or gain

Overall, 12.1 and 10.1 % of participants had attempted to lose or gain weight, respectively *(*Table 2 and 3*)*. Attempts to lose weight were significantly higher (25.7 % [22.4-29.4]) in participants who had positive FID index scores compared to those with negative FID index scores and FID index scores that were equal to zero (6.9 % [5.3-9.0] and 7.3 % [5.7-9.1], respectively) *(*Table 2*)*. On the other hand, attempts to gain weight were significantly higher (19.9 % [17.3-22.9]) in participants who had negative FID index scores compared to those with positive FID index scores and FID index scores that were equal to zero (4.5 % [3.3-6.0] and 6.3 % [4.9-8.1], respectively) *(*Table 3*)*.

**Table 2:** Attempts and methods used for weight loss by body dissatisfaction/Feel Ideal Index (FID), in South Africans aged 15 years or older^a^, SANHANES-1, 2012 [24]

Body dissatisfaction/Feel Ideal Index (FID)	Tried to lose weight during the past 12 months^b^		Weight loss methods used^b^							
			Diet related^c^		Increased physical activity		Used weight reducing medication/supplements/ products		Used other methods^d^	
	n(%)	95 % CI	%	95 % CI	%	95 % CI	%	95 % CI	%	95 % CI
<0: Perceived < Ideal	1965(6,9)	[5.3-9.0]	61,2	[47.4-73.4]	31,2	[19.7-45.7]	10,9	[3.2-31.5]	11,8	[6.3-21.2]
0: Perceived = Ideal	2715(7,3)	[5.7-9.1]	49,5	[38.3-60.7]	40,9	[30.2-52.6]	7,9	[4.2-14.3]	13,3	[6.6-25.1]
>0: Perceived > Ideal	1697(25,7)	[22.4-29.4]	68,6	[60.8-75.4]	40,0	[31.8-48.9]	9,3	[6.3-13.3]	6,6	[3.7-11.7]
Total^a^	6377(12,1)	[10.5-13.9]	62,4	[56.4-68.2]	38,7	[32.3-45.5]	9,2	[6.6-12.8]	9,2	[6.2-13.6]

^a^Participants for whom valid weight and height readings were recorded, and those who answered the questions on perceived and ideal body silhouettes and weight loss

^b^Of those who tried to lose weight. Multiple responses were allowed for this question

^c^Ate less, ate smaller portion sizes, increased variety in the diet, or ate healthier foods

^d^Other methods for weight loss included (but were not limited to) stress, increased water intake, and drinking warm or hot water

**Table 3:** Attempts and methods used for weight gain by body dissatisfaction/Feel Ideal Index (FID), in South Africans aged 15 years or older^a^, SANHANES-1, 2012 [24]

Body dissatisfaction/Feel Ideal Index (FID)	Tried to gain weight during the past 12 months^b^		Weight gain methods used^b^					
			Diet related^c^		Reduced physical activity		Used supplements	
	n(%)	95 % CI	%	95 % CI	%	95 % CI	%	95 % CI
<0: Perceived < Ideal	1965(19,9)	[17.3-22.9]	89,4	[85.0-92.6]	4,8	[2.9-7.9]	6,4	[4.1-9.8]
0: Perceived = Ideal	2713(6,3)	[4.9-8.1]	77,4	[66.3-85.6]	10,2	[5.6-18.0]	16,4	[9.0-28.0]
>0: Perceived > Ideal	1693(4,5)	[3.3-6.0]	83,7	[71.5-91.3]	9,3	[4.2-19.5]	9,1	[3.5-21.3]
Total^a^	6371(10,1)	[8.9-11.5]	85,6	[81.3-89.0]	6,8	[4.8-9.5]	9,3	[6.4-13.3]

^a^Participants for whom valid weight and height readings were recorded, and those who answered the questions on perceived and ideal body silhouettes and weight gain

^b^Of those who tried to gain weight: Multiple responses were allowed for this question

^c^Ate more food, ate fatter portion sizes, limited variety in the diet, or made unhealthy food choices

In relation to the methods used to lose or gain weight, the majority of participants (62.4 and 85.6 %, respectively) adjusted their diet (Table 2 and 3). Moreover, more than a third (38.7 %) and 6.8 % of participants increased or decreased their physical activity, respectively, in order to induce weight loss or to gain (Table 2 and 3). Almost one out of ten (9.2 %) of the participants reported using medication, supplements or other products in order to lose weight. Moreover, “other methods” such as increasing water intake, drinking warm/hot water or stressful circumstances were volunteered as having contributed to the loss of body weight at an equal rate (9.2 %; Table 2). Finally, the use of supplements in order to gain weight was reported by 9.3 % participants (Table 3).

## Discussion

This secondary data analysis investigated, for the first time, different dimensions of BI on a nationally representative sample of South Africans aged 15 years and older using BID and FID index scores, their relationship with body mass index, and the relationship of FID index scores with weight management. The findings showed that while over half (50.8 %) of South Africans were overweight or obese, only 12.1 % reported having attempted to lose weight. Close to half (45.3 %) of the total sample were highly dissatisfied about their BI and 84.5 % had a largely distorted body image. The most preferred weight management methods were adjusting dietary intake and to a lesser extent physical activity.

Despite the afore-mentioned findings, there are some limitations to the current research. These include: i) presentation of information on both adults and adolescent children in the same paper – an issue that could not be avoided given the current SANHANES-1 participant selection procedure, ii) presentation of BI data using descriptive analysis only; iii) exclusion of analytical statistics (such as multivariate models, regression analysis, in particular) and iv) failing to control for potential socio-demographic confounders. However, it has to be noted that further analysis to address these limitations, including logistic regression, has been presented elsewhere.

Taking a closer look to the current findings, it appears as though South Africa is a nation that is either biased in terms of its BI or simply has inaccurate perception of body size, judging from the fact that 84.5 % of South Africans aged 15+ years either under- or over-estimate their body size. These results are corroborated by previous South African regional studies that suggest that adult South Africans misrepresent their body size, with adult women doing so to a greater extent than young women and men [8, 9, 18]. In the current analyses it is not clear whether the large majority of South Africans are purposefully misrepresenting their body size or are honestly not aware of it. What is known is that according to the SANHANES-1 report [24], 96 % of South Africans 15+ years were able to correctly identify a thin or fat body size. As such, it appears as though underweight and obese South Africans may be purposefully misreporting their body size to emulate unrealistic stereotypes of BI. This could be due to the influence of the South African societal norms that either emphasize or denigrate thinness or excess weight and sometimes stigmatize thin or obese individuals [18, 29–33]. In this regard, the available evidence internationally has also shown that social stigma associated with being obese is more prevalent and affects the BI of obese individuals [34, 35]. Kwan and Trautner [35] found that social stigmas attached to obesity, which relate to the attributes and cultural emphasis placed on appearance, especially body size, do impact on body image perceptions. According to the current results, it appears that in South Africa under- or overweight individuals who score negative or positive on the BID index may not be aware of their body size and consequently may not be seeking help for weight-related issues. The latter may have important implications in relation to the increasing prevalence of obesity in the country [8, 24].

Fewer participants attempted to lose or gain weight, despite the high prevalence of over- and underweight, with almost half of the participants (45.3 %) being dissatisfied about their BI (with equal spread between those who desired to be fatter or thinner [21.9 or 23.4 %, respectively]). However, perceiving one-self to be overweight or underweight strongly correlated with weight loss / gain attempts in adult men and women [36].

In the current analysis, irrespective of demographic variables, a significant number of respondents who overestimated their body size also desired to be bigger. Similarly, a significant number of respondents who underestimated their body size also desired to be thinner. This dichotomy of BI concerns was noticeable in both extremes of BMI categories, *i.e.* both underweight and obese. This seems to suggest that in South Africa BMI has an effect on BI distortion and dissatisfaction. Indeed, Mchiza *et al.* [37] have shown that in South Africa BI concerns increase with an increase in BMI, with those individuals having higher BMI tending to underestimate their body size and being dissatisfied by it to a greater extent than those with lower BMI. However, the strength of the current findings is that BI concerns in South Africa were shown to worsen from individuals with normal (acceptable) BMI to individuals presenting with underweight and obesity.

Finally, it has been shown that majority of the South Africans who perceived themselves to be thinner had attempted to gain weight, while those who perceived themselves as fatter had attempted to lose weight. Moreover, fewer South Africans used medication, supplements or unproven methods to manage their weight. These results are in contrast to other South African evidence that suggested that BI dissatisfaction results in the misuse of extreme weight-adjustment measures [18, 30, 38, 39].

## Conclusion

The current results suggest that the majority of South Africans have a largely distorted BI and are dissatisfied about their body size, but BI concerns vary according to BMI. Body mass index is influenced by socio-demography, and as such it appears as if socio-demographic factors have the most effect on BI. Finally, in South Africa the preferred methods of weight adjustment are those that are proven to be effective, *i.e.* adjusting food intake and physical activity [40]. This information opens another window of opportunity for targeted weight education interventions and beneficial weight loss strategies that include healthy eating and increasing physical activity.

## Abbreviations

SANHANES-1: The first South African National Health and Nutrition Examination Survey
BI: Body image
BMI: Body mass index
FID index: the Feel-Ideal-Difference index – suggesting body image dissatisfaction
BID index: the body image distortion
HSRC: Human Science Research Council
PHHSI: Population Health, Health Systems and Innovation

## Footnotes

1 ZAR: South African Rand - exchange rate: 1USD = ZAR 10.50 at the time of manuscript submission
